# Does the good life feel good? The role of positive emotion in competing conceptions of the good life

**DOI:** 10.3389/fpsyg.2024.1425415

**Published:** 2024-08-07

**Authors:** Lukas Novak, Nona Kiknadze

**Affiliations:** Flourishing Lab, Department of Counseling Psychology, School of Education and Human Development, University of Miami, Miami, FL, United States

**Keywords:** flourishing, positive emotion, wellbeing, theory, functioning

## Abstract

Flourishing refers to one kind of generalized wellbeing. Contemporary flourishing research often privileges positive emotion in the theorization and measurement of the construct, such that flourishing is frequently conceptualized as involving a predominance of positive over negative emotions. Positive emotions are thus, on some views of flourishing, seen as an essential component of “the good life.” This paper explores the nuanced variations in conceptions of the good life, focusing on the interplay between positive emotion and flourishing. Through an analysis of contemporary perspectives on flourishing, we underscore the diversity in conceptualizations of flourishing and the implications of this diversity for flourishing theorists. Our review reveals significant disparities in perspectives regarding the significance of positive emotion in the pursuit of a good life. Furthermore, we delineate the theoretical distinctions between objective-list approaches and functional approaches to flourishing, highlighting their respective advantages and limitations. Theoretical dissensus persists regarding whether positive emotion is a necessary constituent of the good life, thus prompting a critical examination of the justification for its inclusion in flourishing models. Finally, we emphasize the need for greater theoretical clarity in defining wellbeing to inform both research endeavors and societal discourse. We suggest that an adequate appreciation of variation in the development and maintenance of flourishing requires admitting for more complex relationships between the construct and both positive and negative emotionality, while embracing the cultural and individual variety that are unavoidable in accurate models of human life.

## 1 Introduction

There are several goods in human life that are almost universally pursued: among these are pleasure, health, and meaning. A life which involves many or all of these goods might be labeled a good life, and the individual living that life might be described as *being well, living well,* or experiencing *wellbeing*. However, as might be expected, there exist various approaches to clarifying what exactly makes a life good. For example, each of the three preceding formulations suggests its own demarcation of what is important to the good life. *Experiencing wellbeing*, by emphasizing experience, orients one toward subjective states, and implicitly establishes a standard for evaluating a life on the basis of those states. *Living well*, on the other hand, through the use of the active verb *living,* emphasizes those components of the good life which involve psychological participation – this phrasing suggests that the good life is a question of one’s actions, and perhaps their relationship to a changing situation. Finally, *being well*, through the passive verb “be,” suggests a global appraisal, which might involve both internal and external factors (e.g., one can “be” lucky, where this luck is a feature of one’s circumstance, and that luck may be an important feature of being well).

Clarifying these differences is more than a linguistic exercise—it demonstrates that subtle variations in phrasing can lead to dramatic differences in the analysis of the good life and the determination of which lives are good. Such differences exist in practice: psychologists studying superlative positive states, such as wellbeing, thriving, and flourishing, show marked diversity in their conceptualizations of the relevant constructs, with varying emphasis on positive experience, positive functioning, and overall felicity (Fowers et al., 2023; Novak et al., 2024). Variation in conceptual approach is often an indication of successful scientific practice, and some diversity in conceptualization reflects an active field engaged in ongoing refinement. However, the extant variation in views of what it means to live well, be well, or have a good life are sufficiently dramatic as to suggest the existence of genuinely different conceptions of the human good.

It is not our goal to resolve those differences here. Instead, we aim to clarify how various notions of the good life are expressed in contemporary views of flourishing, with a focus on the role of positive emotion. While contemporary flourishing research generally privileges the role of positive emotion in the conceptualization of the good life, we will argue that flourishing theorists do not, in fact, agree on the role of positive emotion for flourishing, nor do they agree about the implicit role of external factors, or felicity, in doing well.

Our secondary aim is to explore the implications of various commitments regarding the good life. As will be seen through the example of positive emotion, the meaning and desirability of even a seemingly innocuous indicator of flourishing can be radically changed on the basis of one’s conceptualization of wellbeing, which has both intellectual and practical consequences for the field.

We deliberately refrain from addressing moral foundations in this discussion due to the complex and contentious relationship between moral and developmental notions of flourishing. The debate over whether “good” encompasses moral goodness or solely developmental benefits is nuanced, and is arguably more pertinent to modern perspectives; for example, Aristotle might have deemed this distinction insignificant. Although some theorists maintain that the “Good Life” is inseparable from the “Morally Good Life,” contemporary flourishing research frequently excludes moral considerations. This paper mirrors this contemporary approach by concentrating on flourishing without engaging in moral discourse. An examination of that literature is beyond the scope of this article. For a review of virtue ethics and morality in psychology, see Fowers (2012b).

## 2 Emotions and valence

Before exploring the role of emotion in flourishing theory, we here provide some introductory remarks on emotion and its role in living. Emotions typically refer to complex psychological and physiological states that involve a combination of subjective experiences, physiological arousal, expressive behaviors, and cognitive processes (James, 1884; Schachter and Singer, 1962). These multifaceted phenomena are integral to human experience and play a fundamental role in shaping cognition and behavior (Gross, 2015). Emotions encompass a wide range of affective states, including but not limited to happiness, sadness, anger, fear, and disgust (Ekman, 1999). Emotions are often conceptualized as adaptive responses to internal or external stimuli, serving various functions such as facilitating communication, guiding decision-making, and promoting social bonding (Keltner and Haidt, 2001; Tyng et al., 2017; Šimić et al., 2021). Additionally, emotions can be characterized by their valence (positive or negative) and arousal level (intensity), which contribute to their subjective experience and behavioral outcomes (Russell, 1980).

The scientific literature on emotions delves into two main perspectives: essentialist and constructionist (Boiger and Mesquita, 2012). Essentialist models view emotions as relatively universal affective responses, with Russell’s circumplex model and Ekman’s paradigm of basic emotions being prominent examples. Russell’s model categorizes affective states based on valence (pleasant-unpleasant) and arousal (active-passive), while Ekman’s paradigm identifies six basic emotions: anger, surprise, disgust, enjoyment, fear, and sadness, each associated with distinct neurophysiological systems (Ekman et al., 1987; Posner et al., 2005). However, these models have faced theoretical and empirical critiques, leading to the emergence of constructionist theories, which view emotions as products of social interaction and cultural context (Kövecses, 2003; Gendron et al., 2018). Between essentialist and constructionist perspectives lies a middle ground, integrating elements of both viewpoints. These theories acknowledge the possibility of universal emotional experiences while recognizing cultural influences on emotional expression and interpretation (Feldman Barrett, 2006; Fong, 2006; Matsumoto and Hwang, 2012).

Valence, a key component of emotion theories, involves the classification of emotions as positive, negative, or both positive and negative, based on evaluative responses to one’s environment (Cacioppo et al., 2012). Positively-valenced emotions are associated with pleasure and reward, while negatively-valenced emotions are associated with fear, discomfort, and withdrawal (Cacioppo and Berntson, 1994). Traditionally, theories of wellbeing depict positively-valenced emotions as conducive to wellbeing, while negatively-valenced emotions detract from it (Diener, 2000). However, recent scholarship has complicated this dichotomy by highlighting the nuance of ambivalent emotions, emotional states with encompass both positive and negative valence (Rees et al., 2013). Unlike univalent emotions, which are characterized by a clear valence of either positivity or negativity, ambivalent emotions can involve a combination of contradictory feelings, such as experiencing “nostalgia” or feeling “bittersweet.” Ambivalent emotions challenge traditional conceptualizations of emotions as operating along a unidimensional continuum of valence and highlight the nuanced and complex nature of human emotional experiences. Research indicates that emotional ambivalence can have many positive psychological benefits, such as facilitating creativity, aiding in coping with distressing events, improving decision-making, fostering resilience, and promoting compassion (Hershfield et al., 2012; Rees et al., 2013; Moss and Wilson, 2014; Candiotto, 2023).

This polarity of human emotions is also challenged by enactivist theories which posit that emotions and their valence emerge from one’s embodied engagement with the world rather than being solely products of internal mental processes or external stimuli (Depraz et al., 2003; Colombetti, 2014). The enactive approach recognizes that emotional experiences are multifaceted and context-dependent. In line with the literature on ambivalent emotion, emotions are not simply positive or negative; they vary in intensity, nuance, and meaning based on situational context and personal interpretation. This perspective can provide a more comprehensive framework for studying emotional experiences and their implications for human flourishing.

While we recognize the breadth of cultural, historical, and philosophical perspectives on emotions exceeds the scope of this paper, it is essential to underscore several key examples that contribute to a nuanced understanding of the intricate relationship between emotions and wellbeing. It is also critical to note that most flourishing research conducted thus far has predominantly relied on samples from Western, Educated, Industrialized, Rich, and Democratic (WEIRD) societies and has been led by scholars from similar backgrounds (Henrich et al., 2010). This trend raises concerns regarding the extent to which these findings can be generalized to a broader and more diverse global population (Fernández-Ríos and Novo, 2012; Hendriks et al., 2019). Although our focus within this paper lies in reviewing how current flourishing measures in the psychological literature approach emotion, we also acknowledge that these approaches may inadvertently incorporate WEIRD assumptions about emotions, potentially constraining their relevance and applicability beyond WEIRD contexts.

Diverse theoretical traditions support the perspective that there is also value found in negative emotions. In addition to the recognized benefits of emotional ambivalence, various theoretical traditions advocate for the importance of negative emotions, challenging the prevailing notion that positive emotions alone contribute to wellbeing. Philosophical perspectives, particularly within existentialism, emphasize the significance of negative emotions in shaping human existence. Existentialist thinkers like Sartre and Nietzsche argue that embracing the full spectrum of human emotions, including those deemed negative, are necessary steps towards embracing self-realization and finding meaning in existence (Nietzsche, 1886; Sartre, 1956). According to these philosophies, negative emotions such as despair, anxiety, and anguish are integral to the human condition, prompting individuals to confront fundamental questions about existence and meaning.

Moreover, within certain religious traditions like Buddhism, negative emotions are viewed not as hindrances but as opportunities for spiritual growth and enlightenment. The Buddhist concept of “dukkha,” often translated as suffering or unsatisfactoriness, underscores the inevitability of experiencing negative emotions in life (Lama and Cutler, 2009). Rather than seeking to eliminate negative emotions, Buddhist teachings emphasize understanding their root causes and cultivating equanimity in the face of adversity. Through practices such as mindfulness and compassion, individuals develop awareness of the transient nature of both positive and negative emotions.

The acknowledgment of the possible advantages associated with ambivalent and negative emotions disrupts the conventional division within positive psychology, highlighting the intertwined nature of positive and negative facets of human existence (Ryff and Singer, 2003). This newer perspective, often referred to as the “second wave” of positive psychology, recognizes that wellbeing encompasses a multifaceted interaction among positive, negative, and ambivalent emotional states (Lomas and Ivtzan, 2016; Lomas, 2017). Consequently, gaining insight into the subtleties of emotional valence is a crucial step toward comprehensively examining the impact of emotions on wellbeing and psychological flourishing.

Cultural differences challenge the universality of happiness as a marker of flourishing, with Eastern cultures historically prioritizing equilibrium and harmony over high-arousal positive states such as joy and happiness (Uchida and Kitayama, 2009; Lim, 2016; Lu and Xie, 2021). Cultural disparities also extend to the impact of positive and negative emotions on health outcomes. Miyamoto and Ryff (2011) illuminate the concept of East Asian dialectical thinking, which involves embracing contradictions, such as maintaining a balance between positive and negative emotions, in contrast to the Western inclination to prioritize positive emotions while diminishing the significance of negative ones (Miyamoto and Ryff, 2011; Kitayama and Park, 2017). Furthermore, a comparative study involving Japanese and American samples revealed that Japanese individuals were more inclined to endorse a dialectical perspective on emotions compared to their American counterparts. This inclination was associated with fewer negative health symptoms among Japanese participants (Miyamoto and Ryff, 2011). In Japan, positive affect often fails to predict health outcomes as observed in Western contexts (Kitayama and Park, 2017; Yoo and Ryff, 2019). Similarly, negative affect, commonly associated with poorer health outcomes in the United States, does not exhibit the same predictive pattern in Japan (Miyamoto and Ryff, 2011; Park et al., 2020). These findings underscore the importance of considering cultural norms when examining the experience and health implications of emotions.

Several emerging initiatives recognize the limitations of Western-centric paradigms and aim to incorporate diverse cultural perspectives into flourishing research. Efforts like the Global Wellbeing Initiative seek to broaden the conceptualization of flourishing by introducing items assessing balance and harmony that reflect diverse perspectives of emotional wellbeing into global surveys (Lambert et al., 2020). These endeavors represent crucial steps towards embracing cultural diversity in understanding and measuring flourishing. We urge researchers to continue integrating relevant cultural paradigms into their work for a more comprehensive grasp of human wellbeing. Recognizing the significance of negative or ambivalent emotions, as endorsed by various psychological, philosophical, and religious traditions, provides valuable insights into the intricate realm of human emotional experiences and wellbeing. Rather than exclusively pursuing perpetual positivity, embracing the complexities of mixed valence emotions offers an alternative viewpoint on wellbeing that is often ignored in the current psychological discourse on flourishing measurement. While there is evidence supporting an association between positive emotions and psychological flourishing in some cultural contexts, a comprehensive understanding of wellbeing demands acknowledgment of the intrinsic value in both positive and negative emotional states and necessitates exploring the diverse ways in which culture shapes emotional experiences (Tsai and Clobert, 2019).

## 3 Contemporary wellbeing research

Social scientists employ various terms, such as wellbeing, flourishing, thriving, and happiness, to articulate the concept of leading a fulfilling life (Haybron, 2013). Despite their distinct nuances and historical antecedents, these terms collectively seek to address the fundamental question of what constitutes a life well lived. Haybron’s (2013) foundational taxonomy categorizes wellbeing into three primary domains: positive emotions, life satisfaction, and flourishing. The majority of this manuscript will explore approaches to flourishing, or wellbeing when considered in a superlative sense. That is, for the purposes of this paper, flourishing will be considered as a form of wellbeing which is general, pervasive, and summative—in short, a form of wellbeing that points to a life well-lived. However, we note that wellbeing and flourishing are terms frequently used interchangeably in the literature, with very blurred conceptual boundaries. The purpose of this manuscript is, in part, to explore the precise meaning of various iterations of generalized wellbeing.

Contemporary conceptualizations of living well vary. Some theorists take a hedonic approach, in which wellbeing is understood as the preponderance of positive emotion over negative emotion (Campbell, 1976; Diener et al., 2010). Others take an evaluative approach, in which wellbeing is understood in terms of a participant’s overall assessment of their life (see Diener, 2000). These two approaches can be roughly grouped under the heading of Subjective Wellbeing (SWB)—SWB refers to when an individual has positive thoughts or feelings about his or her life. These approaches have an intuitive appeal, in that positive emotions and appraisals are labeled as integral parts of wellbeing because they are experienced as pleasant and desirable.

In contrast to SWB approaches, other theorists of wellbeing take an objective list approach, in which living well is understood as involving the presence of a variety of goods, which may include life-satisfaction or pleasure, but which may also include things like high-quality relationships, financial security, or a sense of personal engagement (Psychological Wellbeing Scale: Ryff, 1989, Mental Health Continuum: Keyes, 2002, Flourishing Scale: Diener et al., 2010; Flourishing Index: VanderWeele, 2017). These researchers emphasize features like psychological functioning and the content of life, as well as an individual’s experiences, to offer a more expansive vision of wellbeing. Contemporary approaches to flourishing, or generalized wellbeing, tend toward this latter approach, measuring the degree to which an individual is living well using high-level questionnaires that tap into multiple dimensions of living.

Objective list approaches to flourishing suffer from an epistemological weakness, in that they are difficult to refute but easy to replace. Existing objective list approaches to flourishing are more notable for their differences than their similarities, with each offering a vision of what constitutes wellbeing for people in general (Novak et al., 2024). Critiques of these approaches on cultural and idiographic grounds have been made (Mathews and Izquierdo, 2008; Fowers et al., 2023; Kiknadze and Fowers, 2023). One noteworthy feature of these objective list accounts is the frequent central role of positive emotion—despite multiple, significant differences across various conceptualizations of flourishing, positive emotion often emerges as a feature deemed to be essential to the best life.

This emphasis on positive emotion as a central component to the good life has not always been popular, nor is it universally accepted now—earlier attempts to characterize good living emphasized features like psychological health and functioning, which sprang from a different perspective on the role of human beings in their environment. From this alternative perspective, living well was less about attaining very positive feelings, and more about adapting to an environment that posed various developmental challenges (Jahoda, 1958).

The role of positive emotions in a given conceptualization of flourishing offers a useful foil for analyzing the sort of flourishing that is being studied. In many objective-list approaches, positive emotions are an ultimate good, and possess a simple and constitutive relationship to flourishing; in short, it is good to feel good. On others, flourishing conceptually depends on functioning well, and emotions are part of the good life when they are *adaptive,* i.e., inasmuch as they are *appropriate,* or *functional* (e.g., Ryff, 1989 or Vittersø, 2016).

The two preceding perspectives are frequently conflated without proper examination in contemporary models of flourishing. This is unfortunate because there are situations in which these approaches provide contradictory intuitions for which situations constitute living well. For example, it is possible that maximizing positive emotion can interfere with functioning; in these situations, we as researchers are presented with a dilemma on what, precisely, should be deemed a desirable state of being. These situations recall Mill’s famous quip: “It is better to be a human being dissatisfied than a pig satisfied,” which we might rephrase as “It is better to function well without positive experience than to function poorly with positive experience” (Mill, 2008).

To clarify the role of positive emotion in contemporary flourishing models, we briefly introduce and analyze the role of positive emotion in seven prominent models of flourishing, or generalized wellbeing, identified in a recent review (Novak et al., 2024). To add depth to the perspective afforded by the analysis, we include two highly influential historical models of generalized wellbeing After this brief review, we summarize dimensions along which various theories of generalized wellbeing differ and consider the implications of these differences for research and practice.

## 4 Objective-list approaches to flourishing: positive emotion as a primitive good

Some contemporary models of flourishing take an additive, multi-dimensional approach to the construct. Per these perspectives, flourishing denotes an optimal positive condition characterized by a life that actively engages with and maximizes attainable goods. This perspective can be described in terms of a bucket, or a list—life is like a bucket, and the more goods that are in the bucket, the better the life is, with relatively little consideration for how those goods might interact. These goods can be both internal and external. An internal good might be positive emotion, and external good might be financial security. Importantly, the status of these goods as goods is rarely questioned in these models—they are deemed as contributing to flourishing across all circumstances. Thus, inasmuch as positive emotion is deemed one of the constituent goods of flourishing, one flourishes more to the extent that one experiences more positive emotion. Furthermore, the relationship of positive emotion to flourishing is primitive—it is simply posited as a universal good, and one of the core components of the good life. This view can be seen in the following measures of flourishing, which will be examined in partial detail.

### 4.1 Huppert and So’s flourishing measure

When introducing their measure of flourishing, Huppert and So (2013, p. 838) define the construct as: “the experience of life going well,” and “a combination of feeling good and functioning effectively.” They clarify that flourishing is “synonymous with a high level of mental wellbeing,” and that it “epitomizes mental health” (2011, p. 838). Thus, from the outset Huppert and So take an explicit commitment which binds flourishing with positive emotion, or “feeling good”—positive emotion is seen as the *expression* of flourishing.

In order to develop their measure, Huppert and So (2013) select 10 items from the European Social Survey. Their 10-item measure has 10 subscales, each measuring a feature which they believe to be important to flourishing. These features are: positive emotion, competence, emotional stability, engagement, meaning, optimism, positive relationships, resilience, self-esteem, and vitality. These features were developed through an investigation of which emotional and psychological features were the opposites of psychological features experienced by individuals with depression and anxiety.

In establishing a diagnostic cut-off for flourishing, the authors cluster their 10 subscales into three broad categories: positive characteristics (emotional stability, vitality, optimism, resilience, and self-esteem), positive functioning (engagement, competence, meaning, and positive relationships), and positive emotion. Interestingly, the authors argue that the positive emotion item correlates strongly with an index of life-satisfaction, and that this item alone reflects an individual’s appraisal of their life; this is taken to be a rough index of their hedonic wellbeing. The authors create a variety of rules by which an individual can score as flourishing, but most importantly for our argument, they assert that an individual *must* report more positive emotion than the median survey respondent in order to flourish.

Huppert and So thus articulate a vision of flourishing with positive emotion at its center. The goodness of good feelings is unquestioned—good feelings are in themselves constituent of flourishing—and an individual who does not feel more positive emotion than the median participant cannot be said to flourishing, regardless of functional considerations. For example, an individual could be functioning extremely well, with superb scores on all 9 sub-scales of positive functioning and positive characteristics, but, due to some unfortunate life event, feel quite sad for some time. Per Huppert and So, this individual is not flourishing. This is a clear example of the objective-list approach to flourishing which posits positive emotion as a primitive and necessary good in the good life.

### 4.2 The mental health continuum

Keyes (2002), like Huppert and So, identifies flourishing as a combination of both positive functioning and positive feeling. He labels flourishing as the presence of generalized mental health, where mental health involves three distinct forms of wellbeing: emotional wellbeing, or the presence of positive attributions and affect; psychological wellbeing, or generally positive psychological functioning; and social wellbeing, or generally positive social functioning. In his expression of the importance of functioning for flourishing, Keyes shares a significant conceptual vocabulary with the authors who will be introduced later in this article. However, he makes a strong commitment to positive emotion when clarifying the diagnostic criteria for his conception of flourishing. In his sub-scale of emotional wellbeing, Keyes includes two measures, one for positive affect, and one for general life satisfaction. In order to qualify as flourishing, Keyes requires that an individual score in the upper-tertile on at least one of these two measures of emotional wellbeing. Thus, Keyes (2002, p. 210) binds flourishing up with experiencing more than average amounts of either positive emotion or life-satisfaction. In this view, positive emotion is regarded as a fundamental and indispensable element of “complete mental health”. Our previous example, of a sad but highly functioning individual, again does not meet the criteria for flourishing due to the absence of positive emotion. This illustration underscores the similarity between Keyes’ framework and the approach advocated by Huppert and So.

### 4.3 The comprehensive inventory of thriving and the flourishing index

Su et al. (2014) develop a model and measure of generalized psychological wellbeing and holistic positive functioning which has seven dimensions: subjective wellbeing, which includes life satisfaction and positive feelings, positive relationships, engagement, meaning and purpose, mastery, autonomy, and optimism. To justify their dimensions, they cite a handful of recent flourishing theorists, but assert that subjective wellbeing, in the form of positive feelings and life satisfaction, is a key component of positive functioning. This position is relatively unquestioned. Consequently, they adopt a stance positing that positive emotion is intrinsic to living well, regardless of an individual’s level of functioning, and can be considered as offering an alternative objective list approach to flourishing, alongside Keyes and Huppert & So.

VanderWeele (2017, p. 8149), when describing a new model and measure of flourishing, states: “I would argue that, regardless of the particulars of different understandings, most would concur that flourishing, however conceived, would, at the very least, require doing or being well in the following five broad domains of human life: (i) happiness and life satisfaction; (ii) health, both mental and physical; (iii) meaning and purpose; (iv) character and virtue; and (v) close social relationships”. VanderWeele goes on to include financial stability as a sixth component (Secure Flourishing Index: Węziak-Białowolska et al., 2017). Like the previous scholars, VanderWeele emphasizes the pivotal role of positive emotion in relation to flourishing. According to his perspective, an individual who is mentally healthy but experiences negative emotion is considered to fare worse compared to someone who experiences greater levels of positive emotion, irrespective of other aspects of his or her life situation.

Each of the preceding four models of flourishing establishes positive emotion as a core component of the construct (Mental Health Continuum: Keyes, 2002, Flourishing: Huppert and So, 2013, Comprehensive Index of Thriving: Su et al., 2014, and Flourishing Index: VanderWeele, 2017). The relationship between positive emotion and flourishing is, across the four models, relatively unquestioned. Instead, positive emotion becomes an unconditional good, alongside the likes of positive functioning, and in the case in which an individual feels negative emotion, they are accordingly flourishing less.

As an important counterpoint to these approaches, we can introduce a model and measure of flourishing which retains the objective-list approach to the primitive goods which make up the good life, but does not include positive emotion among those goods.

### 4.4 The flourishing scale

Diener et al. (2010, p. 144) developed their scale of flourishing to “complement existing measures of subjective wellbeing.” They note the need for brief and effective flourishing measurement, and link their scale to the work of Ryff, Singer, and Ryan and Deci (Flourishing Scale: Diener et al., 2010). Their approach is explicitly functional, and they claim that the sense in which they are measuring flourishing transcends the psychological, which is to say, that they seek to understand living well not only in terms of psychological states, but also in terms of positive external goods. Their scale has eight inter-related categories, each deemed to be essential for flourishing: a sense of meaning, supportive social relationships, engagement, contributing to the happiness of others, competence, a sense of being a good person, optimism, and being respected by others. What is fascinating about this conceptualization of flourishing is the inclusion of items which are entirely outside of the scope of psychological functioning—say, *being respected.* In this case, Diener et al. have effectively taken an objective-list approach, in which flourishing consists of a set of goods which are primitively related to living a good life; their approach is distinct from the other objective-list approaches reviewed here, though, in their exclusion of positive emotion from that list of primitive goods.

What we can conclude from the previous models, taken together, is that one dominant lens through which to understand the ‘good life’ is the objective list approach, in which the good life consists of primitive goods, both internal and external. This good life can involve psychological functioning, but might also involve positive emotions, external luck, or other features. Importantly, there are disagreements among theorists as to the role of positive emotion in the life which participates in primitive goods—some, like Huppert and So, take a strong position, in which a life without more than the median amount of positive emotion cannot be said to be a flourishing life, while others, like Diener et al., do not even include positive emotion among those primitive goods which make a life good.

## 5 Functional approaches to flourishing

Theorists of flourishing which emphasize psychological functioning focus more on *living well* than *being well altogether*; they rely on a critical, meta-psychological position which can be labeled the ‘function argument.” The function argument holds that living well involves fulfilling characteristic functions; this position was maintained by Aristotle and continues its life among multiple contemporary flourishing theorists and philosophers (Fowers, 2012a; Vittersø, 2016). On this view, emotions are experienced and expressed as part of human functioning. To function well, then, is to experience the appropriate emotions, and no emotions take an exclusive and privileged position when it comes to flourishing. These views often allow for a more dynamic inter-relationship between an individual and their environment in determinations of flourishing. For examples of the functional approach, we examine two contemporary perspectives.

### 5.1 Ryff’s psychological wellbeing

Ryff introduced her seminal model of psychological wellbeing to address what she perceived as a deficiency in comprehensive theorization regarding the construct (Ryff, 1989). She observes that happiness has received disproportionate attention as an indicator of positive psychological functioning, while alternative viewpoints have been neglected. Drawing on the works of theorists such as Rogers, Maslow, Jung, Allport, Buhler, Erikson, Neugarten, and Jahoda, Ryff articulates an alternative conception of psychological wellbeing that places greater emphasis on human capacities and less on positive emotion (for all citations see Ryff, 1989). Her model comprises six components: self-acceptance, positive relationships, autonomy, environmental mastery, purpose in life, and personal growth. Notably absent from this list is emotion—Ryff’s perspective suggests that individuals can achieve psychological wellbeing if they fulfill certain characteristic functions, irrespective of their emotional state. This perspective contrasts with those of Keyes, Huppert and So, and VanderWeele. Unlike these theorists, Ryff posits that a highly functioning but sad individual can still be psychologically well, where that sadness does not detract from their wellbeing. Of significance for our analysis is Ryff’s linking of positive psychological functioning with wellbeing. In doing so, she highlights a distinct form of wellbeing, more akin to *living well in one’s situation* than *experiencing all possible goods in a superlative sense*. This raises intriguing questions about the nature of wellbeing, particularly regarding the possibility of experiencing wellbeing in the presence of difficult emotions such a unhappiness and sorrow—questions that will be further explored in our discussion.

### 5.2 Waterman’s questionnaire of eudaimonic wellbeing

Waterman et al. (2010) developed their model of Eudaimonic Wellbeing (EWB) in response to criticisms that the construct of EWB was becoming vague and ungrounded in any theoretical perspective; to correct for this, they ground their model of EWB, and the instrument measuring EWB, in a close reading of Aristotelean philosophy paired with certain modern interpretations of that philosophy (Norton, 1976; Bartlett and Collins, 2011). The model they develop has six inter-related categories which are thought to comprise EWB—self-discovery, perceived development of one’s best potentials, a sense of meaning, investment of effort in pursuit of excellence, intense engagement, and a sense of activities as personally expressive (paired with enjoyment of those activities). Positive emotion is almost entirely absent from their functional model, with the exception of the final category, in which positive functioning is understood to include a sense of pleasure from what is personally expressive. This caveat creates a distinction from thinkers like Keyes who value positive emotion in an unqualified way—for Waterman, it is only positive emotion as experienced in connection to a particular function that counts for overall wellbeing.

To complement the provided review of contemporary perspectives, we here briefly touch on two highly influential accounts of wellbeing which both adopt a functional perspective.

### 5.3 Aristotle and eudaimonia

Over 2000 years ago, Aristotle put forward a model of human functioning and flourishing which continues to exert influence to this day; it is from this work that we derive the term *eudaimonia*, and an active group of researchers aim to characterize flourishing while remaining loyal to Aristotelean thought (Fowers, 2012a, 2016, 2017; Kristjánsson, 2019). In his Nicomachean Ethics, Aristotle conceived of humans as having characteristic functions, the fulfillment of which constituted the best life (Bartlett and Collins, 2011). Fulfilling one’s functions well across a variety of life’s situations requires use of virtues, which are stable, cognitive and emotional dispositions to act in pursuit of some good. To flourish, per Aristotle, is to be virtuous, or to live well (to live *eudaimonically*); this can be termed in the eudaimonist position, which has both theoretical and empirical facets (see Snow, 2008, for an in-depth, modern defense of the eudaimonist position). Notably, Aristotle did not put much stock into the significance of positive experience, and he certainly believed in the importance of luck, or favorable environmental circumstances, in the best life. Because Aristotle did not emphasize positive emotions, there is the possibility of misunderstanding flourishing due to modern bias. To Aristotle, flourishing simply *is* the enaction of virtues, which constitute, across various domains, the appropriate or best ways to be—thus flourishing is not a subjective state but an objective concordance with an external order, prescribed by development and nature.

### 5.4 Jahoda and complete mental health

In her groundbreaking *Current Concepts of Positive Mental Health*, Jahoda (1958) put forth a model of positive functioning which has since been heavily cited by theorists of flourishing. Jahoda articulates six functions, or capacities, which were, on her view, present in most theoretical work seeking to understand positive psychological functioning: attitudes toward the self, self-actualization, personality integration, autonomy from social influences, an adequate perception of reality, and reasonable mastery over the environment. In creating this list, she cites thinkers from a largely psychoanalytic background, including Allport, Erikson, and Maslow (for full list of citations, see Jahoda, 1958) Jahoda does not, in her model, consider positive emotion to be essential to mental health, and her theory relies more extensively on the concepts of adequate psychological functioning and reality-orientation.

## 6 Overview of approaches to flourishing

We have briefly touched on 9 approaches to wellbeing, flourishing, or “complete mental health,” two of which are influential historical accounts and seven of which are (roughly) contemporary. A survey of these perspectives leads to an appreciation of certain key commitments that each theorist makes when developing a model of the good life. The first, and most salient, dimension involves the grounding of the good life in human functioning (function-approaches) or the grounding of the good life in primitive goods (objective-list approaches). The second dimension, and one which is important for our discussion, is the relative importance of positive emotion for the good life. Objective-list approaches can include positive emotion as a primitive and necessary good for the good life (Keyes), or exclude positive emotion altogether (Diener). Functional approaches can entirely ignore the role of positive emotion (Jahoda), or integrate positive emotion with respect to a specific psychological function (see Waterman’s integration of enjoyment due to personal expressiveness). Finally, both objective-list and functioning approaches can differ in the extent to which they emphasize internal versus external goods. Waterman’s model of eudaimonic wellbeing mentions the external world relatively less when compared to Diener’s model of flourishing, which would require being *respected* by others, or VanderWeele’s model of flourishing, which requires financial security.

These three dimensions of variation allow for an efficient categorization of the nine models of wellbeing (see Table 1). What can be seen from this categorization is a relatively higher emphasis on the importance of positive emotion from objective-list approaches to living well, as opposed to functional approaches. We now briefly turn to the field of emotion regulation to consider current perspectives on the role of positive and negative emotions in human life.

**Table 1:** A brief taxonomy of 9 models of generalized wellbeing, per theoretical commitments.

Model	Functional or objective-list	Positive emotion as essential	Relative focus on internal goods, external goods, or both
Aristotle’s *eudaimonia*	Functional	Not essential	Both
Jahoda’s Complete Mental Health (1958)	Functional	Not essential	Internal goods
Ryff’s Psychological Wellbeing (1989)	Functional	Not essential	Internal goods
Waterman’s Eudaimonic Wellbeing (2010)	Functional	Essential as part of a limited function (expressiveness)	Internal goods
Diener’s Flourishing Scale (2010)	Objective-list	Not Essential	Both
VanderWeele’s Secure Flourishing Index (2017)	Objective-list	Essential	Both
Huppert & So’s Measure (2013)	Objective-list	Essential	Internal goods
Su et al.’s Comprehensive Inventory of Thriving (2014)	Objective-list	Essential	Internal goods
Keyes’ Mental Health Continuum (2002)	Objective-list, with added emphasis on positive functioning	Essential	Both

## 7 Positive emotion and emotional regulation

The field of emotion regulation is expansive, dynamic, and contentious. Decades of research have yielded a wealth of insights, but considerable disagreement persists regarding the central constructs under consideration (For in-depth reviews of the field of emotion regulation, see Gross and Thompson, 2007 and Gross, 2015). Two influential theorists summarize the situation pithily when they say: “It is widely agreed that *emotion* refers to a collection of psychological states that include subjective experience, expressive behavior (e.g., facial, bodily, verbal), and peripheral physiological responses (e.g., heart rate, respiration). It is also widely agreed that emotions are a central feature in any psychological model of the human mind. Beyond these two points of agreement, however, almost everything else seems to be subject to debate” (Gross and Feldman Barrett, 2011, p. 9).

Despite such controversy, in recent years, many theorists of emotional regulation have taken a turn toward the concept of functional emotion—emotions are relatively appropriate, or adaptive, depending on a given situation (e.g., Izard et al., 2008). It is not always clear what adaptive, or appropriate, means, but reference is often given to generalized functioning, and sometimes to wellbeing; frequently, emphasis is placed on the concept that negative and positive emotions both have a role in the functional life (e.g., Kobylińska and Kusev, 2019). Consider the following statements, all made by influential theorists of emotional regulation (emphasis added):

- “Emotional preferences should hinge on the goals people are inclined to pursue. We have not given due consideration to the task of *identifying which emotions are functional and at what levels of intensity and type of expressiveness. Sometimes negative, unpleasant emotions can be more useful than positive emotions.*” (Kashdan and Rottenberg, 2010, p. 866)
- “The goal of the regulatory process is to reach optimal levels of emotion dynamics, so that *emotions can facilitate appropriate responding to the ever-changing demands of the environment*.” (Aldao, 2013, p. 155)
- “The value of the concept of emotion regulation is as a tool to understand how emotions organize attention and activity and facilitate strategic, persistent, or powerful actions *to overcome obstacles, solve problems, and maintain wellbeing* at the same time as they may impair reasoning and planning, complicate and compromise interpersonal interactions and relationships, and endanger health. *It is not the valence of an emotion but the complex processes by which emotions relate to cognition and behavior and ultimately developmental outcomes* that must be conceptualized and studied.” (Cole et al., 2004, p. 318)

These claims highlight the complexity of determining whether an emotion should be deemed as desirable, or, in other words, whether emotional regulation can be deemed “successful.” Importantly, reference is consistently made to an alternate good, whether it be usefulness, responsiveness to the environment, or “developmental outcomes,” which can be used to determine the successfulness of emotional regulation and the desirability of an emotion; notably absent from these comments is an impression of positive emotion as a primitive good, desirable in itself. However, while emotional regulation theorists articulate a relatively common understanding of the importance of both positive and negative emotions for the good life, the alternative good by which they justify that importance is often unspecified, and that “reference good” often differs between accounts and theorists. For example, some theorists cite wellbeing, others functioning, and still others usefulness as indexes of which emotions are desirable and when.

One provocative consequence of theorists who cite wellbeing as an index by which we can assess the appropriateness of an emotion can be seen when we link such a claim with a theory of wellbeing which posits positive emotion as a primitive good (i.e., Keyes’ Mental Health Continuum). In this case, we encounter a sort of chicken-and-egg explanatory circularity—positive emotions, presumably, are suitable because they contribute to wellbeing, but an individual is well precisely because they have positive emotions. This circularity reveals that, when a theory of wellbeing posits positive emotion as a primitive good, it is not explanatory to argue that positive emotions are functional because they contribute to wellbeing, but instead tautological.

What can certainly be seen among emotional regulation theories is a demand for explanation—explanations as to how emotional regulation succeeds, and to why individuals regulate their emotions, especially in the context of supra-hedonic goals. This demand for explanation is not compatible with objective-list approaches to wellbeing which posit positive emotion as a primitive good, because on these models, positive emotion is desirable in itself, without reference to another good. Instead, a demand for an explanation of which emotions are adaptive or appropriate quite naturally blends with functional approaches of wellbeing which ground what is considered good in considerations of characteristic human capacities.

## 8 Discussion and conclusion

In conclusion, our review of seven contemporary and two historical models of flourishing aimed to evaluate their perspectives regarding the significance of positive emotion in the pursuit of a good life. We have identified several theoretical questions that yield substantial variations in conceptions of wellbeing, including the grounding of claims about what constitutes the ‘good life’. Some theorists adopt objective-list approaches, positing a collection of primitive goods, while others advocate functional approaches that ground wellbeing in the fulfillment of characteristic human functions. Furthermore, theories of flourishing may differ in their emphasis on internal versus external goods. Theories of flourishing that emphasize external goods naturally orient our attention toward external factors, and encourage the appreciation of the role of the situation in allowing or not allowing flourishing—theories of flourishing that emphasize internal goods orient our attention toward an individual’s psychological resources, and encourage the appreciation of the role of the individual in adequately *responding* to their situation.

Ethically, the emphasis on external goods raises concerns about equity and justice, as it suggests that individuals’ flourishing is contingent upon factors beyond their control, such as social structures and external circumstances through limitations of birth, or through significant life events like experiencing illness or injury. This perspective implies that individuals facing systemic barriers may struggle to achieve flourishing despite possessing internal resources, leading to potential disparities in wellbeing based on socioeconomic status, race, able-bodiedness, or other factors. In contrast, theories emphasizing internal goods can be used to suggest that everyone has the potential to achieve flourishing despite external circumstances. However, this perspective may overlook systemic injustices and fail to address the structural barriers that prevent certain individuals from accessing the resources needed to flourish.

Finally, we have noted that theories of flourishing can vary in the extent to which they privilege positive emotion as a necessary constituent of wellbeing. In general, objective-list approaches were seen as more likely to argue for positive emotion as a primitive good, while functional approaches are less likely to consider the valence of emotion and more likely to consider the role of emotion in general functioning. This last point can be complicated through an examination of the emotional regulation literature, in which multiple theorists call for conceptual explanations of when and why emotions are appropriate or not appropriate for a given situation (consider a funeral, or the death of a friend, for salient examples). These calls for conceptual explanation are incompatible with objective-list approaches which posit positive emotion as an unexplained good.

These distinctions are important in part because of the public salience of the construct of wellbeing. Wellbeing is almost universally hailed as a desired good, and many public policy initiatives assess success at least partially in terms of wellbeing, or flourishing. However, our review has revealed that what counts as wellbeing can differ dramatically depending on one’s theoretical perspective. For instance, consider the case of a physician dedicated to providing healthcare to marginalized communities. Despite experiencing short-term negative emotions due to long hours and a hectic schedule, this doctor may be actively pursuing valuable long-term goals and providing important services to society. This scenario poses a philosophical inquiry into whether such an individual would be considered “flourishing” according to different theoretical frameworks. In some models, individuals who are highly functional, externally successful, and yet who experience many negative emotions may be deemed as flourishing or living well (e.g., Aristotle, Ryff), whereas in others, these individuals would not be deemed as flourishing (e.g., Keyes, Huppert & So).

There are, of course, certain practical advantages and disadvantages to various commitments about flourishing. Consider the distinction between objective-list and functional approaches—objective-list approaches have the privilege of not needing to ground what is desirable in other desirable things, and thus avoid the difficult question of *why* their list has certain contents and not others. This advantage comes with the simultaneous disadvantage of being unable to refute alternative lists, which is perhaps one of the reasons why multiple objective list accounts, with different lists, exist in the flourishing space today; each lacks the conceptual resources to critique the others. Functional approaches, on the other hand, must complete the arduous task of defending the function argument, and most of the accounts reviewed here simply renege on that responsibility. However, after that task has been satisfactorily (or not satisfactorily) completed, functionalists have a conceptual vocabulary with which to develop rich and comparable theories of wellbeing.

As another arena of practical advantages and disadvantages for the theorist of wellbeing, consider the role of external events in wellbeing. The fact that luck may play a large role in determining whether an individual flourishes is distasteful to some—such a fact can be diminished, or eliminated, through a theory of flourishing that focuses on internal goods. Such a theory would suffer, however, in the inability to address the salient and commonplace intuition that what happens outside of an individual’s mind can bear on the quality of that individual’s life.

Finally, we can assess the practical consequences of the role (or non-role) of positive emotion in overall wellbeing. Theories like Ryff’s, which omit mention of positive emotion and instead reference function, effortlessly address situations in which individuals can live well despite experiencing negative emotion; such views concord with contemporary philosophical work highlighting the epistemic value of negative emotion (Brady, 2018). However, such theories must contend with an alternate objection, which is that many people feel that the good life should involve feeling good; if a theory of the good life omits positive emotion, how is that intuition to be justified? Theories like Keyes’ avoid such a challenge by offering positive emotion a central role, but then must justify the central role of emotion in their theories.

We further observe that it is likely that, on many different conceptualizations of the good-life, varying cultural and individual situations create conditions for variations in the manifestation of flourishing, where that flourishing does not always include a simple preponderance of positive emotion over negative emotion. This observation suggests that extant cultural variation in conceptualization of the flourishing life (e.g., variation in the role of community life in determining wellbeing) demands increased theoretical complexity and flexibility when modeling the relationship between flourishing and emotion, at least for some objective-list approaches (Mathews and Izquierdo, 2008).

Our review reveals that there are no simple and easy answers to the difficult questions facing a theorist of wellbeing. However, we have made it clear that there exists substantial theoretical dissensus about the role of positive emotions in the good life, and in general about which qualities of an individual’s life should be used to ground the claim that their life is good, or that they are living well. We further argue that objective-list approaches to flourishing, which posit positive emotion as an unexplained good, are relatively incompatible with demands for explanation about the appropriateness of emotion found in the emotion regulation literature. A possible resolution to the tension encountered between objective-list and functional approaches to the role of emotion in the good life could involve a reconceptualization of positive emotion as more of an indicator than an outcome; a sketch of this view would involve positing that, in typical cases, positive emotion serves to *indicate* that some good is being accomplished, and that it is not the emotion, but the indicated good, which confers upon positive emotion its desirable quality. This view recognizes the importance of positive emotion without affording it undue and indefensible centrality in a theory of the human good. This view can be critiqued on the grounds that it posits that in certain situations, such as when a good is not being accomplished, it is appropriate to feel negative or neutral rather than positive emotions. Contained in this critique is a conception that the best human life feels the best, while contained in the alternative view is a conception that the best human life is one which most participates in human goods. Resolving these competing conceptions requires further investigation, yet it is evident that any theory of flourishing must, implicitly or explicitly, adopt a position on this issue. Consequently, theories of flourishing that adopt different positions will be relatively incomparable measures.

We conclude by noting that there is a need for greater theoretical clarity with respect to flourishing in order to ensure that the public, and fellow researchers, are informed and prepared for the implications of adapting these models to their research and lives.

## Author contributions

LN: Writing – original draft, Writing – review & editing. NK: Writing – original draft, Writing – review & editing.
